# Strategies to prepare hospice providers to interact with adolescents with a parent in hospice

**DOI:** 10.1017/S1478951525000446

**Published:** 2025-05-30

**Authors:** William Grayson, Denice Kopchak Sheehan, Pamela S. Stephenson, Kristen DeBois, Caitlin Sheehan

**Affiliations:** 1 Department of Nursing, Aya Healthcare Inc, San Diego, CA, USA; 2 Department of Nursing, Kent State University, Kent, OH, USA; 3 Department of Public Health, Muskingum University, New Concord, OH, USA

**Keywords:** Hospice, adolescents/youth, parental death, qualitative research, end of life, palliative care

## Abstract

**Objective.:**

This study aimed to explore what is important for hospice providers to know when a seriously ill parent has adolescent children.

**Methods.:**

The sample included 18 young adults (18–28 years old) whose parents died in hospice or palliative care while they were adolescents (12–18 years old). Semi-structured interviews were conducted virtually via Microsoft Teams. The interviews were audiotaped, transcribed verbatim, and analyzed using a thematic analysis. Themes emerged from the data and were determined by consensus.

**Results.:**

The participants described a variety of skills that are important for hospice providers to know. They provided specific suggestions for hospice providers who seek to help adolescents navigate this critical time when their parents are dying or have recently died.

**Significance of results.:**

These results can also be used to inform the development of interventions that assist hospice providers with strategies tailored to an adolescent’s specific needs. Future research should investigate these topics with a larger, more diverse sample.

## Introduction

In 2023, the National Hospice and Palliative Care Organization (NHPCO) estimated that approximately 236,041 adults within adolescent parenting years (between 35 and 64) received hospice care (NHPCO facts and figures | 2023 edition 2023). Hospice care is intended for people who have less than 6 months to live, yet the median length of stay for hospice patients dropped from 18 days in 2020 to 17 days in 2023 (Mann et al. 2024; NHPCO facts and figures | 2023 edition 2023). Researchers found that the median time for the first conversation about end-of-life (EOL) care was 33 days before death (Mack et al. 2012). Although some parents live with incurable illnesses over an extended period of time, parents with a shorter life expectancy and length of stay in hospice care have less time to prepare for the EOL potentially limiting the extent to which families can derive the full benefits of the program (Punziano et al. 2017; Sutherland 2019). To help adolescents cope with distress better, hospice clinicians stress the need to provide honest information to teens and avoid false hope (Alvariza et al. 2017; Punziano et al. 2017) yet often feel ill-equipped to have conversations with this age group (Fearnley and Boland 2019). It is imperative for parents to understand information about the ill parent’s impending death and come to terms with it before they can talk with their children (Sheehan et al. 2014).

Scholars recommend that these conversations begin with the hospice providers using an open, honest, and clear approach when talking with the parents or the significant adult caregiver of the adolescents to determine the best ways to support them to talk with the adolescents using a family-centered approach to ensure that everyone is being told the same information (Bergersen et al. 2022; Mayo et al. 2016; Punziano et al. 2017; Sutherland 2019; Tranberg et al. 2022).

There is a paucity of research regarding communication between adolescents, defined as 12–19 years of age (Sappenfield et al. 2024), and hospice providers, despite recent efforts to better understand this population’s needs (Tulpin et al. 2024). Much of the existing research relies heavily on the experiences of parents with terminal illnesses but not on the experiences of their minor children (Li et al. 2024). Adolescent children, as they age, are increasingly capable of understanding and empathizing with the suffering their parents may endure (Tulpin et al. 2024). This empathy and ability to further understand their parents’ struggles can generate feelings of anger, injustice, existential sadness, and a worry of future loss (Tulpin et al. 2024). This can also hamper their personal development, and other facets of adolescent life, such as academic and social activities, struggles that can persist in adulthood, having long-term psychological effects (Tulpin et al. 2024). The discussion of serious illness and preparing for the process of dying can be especially difficult for the dying parent and their adolescent children (Mayo et al. 2016). Researchers have found that parents with a terminal illness worry about the effects of their illness on their children’s growth and development, as well as their own ability to continue to spend meaningful time with their children (Li et al. 2024; Sheehan et al. 2016).

Tranberg et al. (2022) found that most EOL conversations happen in the months and days before death while current oncological medicine guidelines (Gilligan et al. 2017; Levy et al. 2014) recommend that conversations regarding EOL should take place earlier, years to months before death. Given the data available, there is no one singular way in which adolescents experience the grieving process (Farella Guzzo and Gobbi 2021). Therefore, one adolescent’s needs may differ greatly from the needs of another adolescent of similar age. Sveen et al. (2016) found that most adolescents do not talk with friends or at school with peers, citing multiple concerns including not knowing how to talk about it, and with whom, and fears of being treated as different. Some adolescents sensed they had not been told about the seriousness of the illness, which led to feelings of being left out by parents and the hospice team. Hospice care providers are uniquely positioned to assess the needs of adolescents with a parent in hospice and develop a plan to include them as valuable members of their parents’ care team.

According to the Institute of Medicine (IOM, 2015), the hospice model is based on a person-centered, family-oriented care provided by an interdisciplinary team designed to support the person and the family as central members of the team. Mayo et al. (2016) found that adolescents with a parent in hospice are not widely acknowledged as a part of the care team, a finding that is contrary to the IOM’s definition of the hospice model. Regular meetings between the hospice care team, the patient, and the family are essential to providing quality EOL care, as the needs of these groups can change over time (IOM, 2015). Further evaluation of the hospice care team is needed to include these integral family members, as hospice care requires flexibility and individual tailoring over time, reflecting patient and family priorities and preferences. Without acknowledgement and involvement of this often-overlooked group of family members, healthcare providers may not fully realize their needs. This study explored the needs of adolescents with a parent in hospice retrospectively with a sample of young adults (YAs) whose parents died when they were adolescents. The objectives of this subset of the larger study were to describe specific strategies that hospice providers can use to help adolescents with a parent in hospice and to elucidate ways to continue to support them after the parent’s death.

## Methods

This study is a subset of the larger study “Interactions Among Adolescents with a Parent in Hospice and the Hospice Care Team.” After receiving approval from the Kent State University Institutional Review Board, 18 YAs (18–30 years old) whose parents died when they were adolescents (13–18 years old) were recruited.

Purposive sampling was used to recruit potential participants from the sample in a previous study (NR012700) via email. Twenty-one YAs met the inclusion criteria from this group. Three declined to participate but did not give a reason. Recruitment strategies were expanded to include flyers throughout a public University, presentations to students, and snowballing.

Two members of the research team (DS and PS) contacted potential participants to respond to their questions and schedule an individual virtual cognitive interview about the reliability and validity of the Coping and Communication Questionnaire (CCQ) on Microsoft Teams. Consent was obtained from the YAs via Qualtrics. The interviews lasted from 60 to 90 minutes between October 2021 and September 2022, 2–15 years after the parent’s death. The participants were invited to turn their camera off, although most left their camera on during the interview, allowing the interviewer to monitor verbal and nonverbal signs of distress; none were noted.

The participants completed the CCQ on Qualtrics. The interviewers asked participants about the clarity, redundancy, and ordering of the questions. The participants went on to describe their experiences from the time their parents were diagnosed with a life-threatening illness until the day of the interview. Each participant was given a $50 Amazon e-gift card as compensation for their time and expertise. Peer debriefing among the two interviewers occurred immediately after each interview. The interviews were digitally recorded and transcribed verbatim; the interviewers reviewed and verified the accuracy of each transcript.

The research team included two doctorally prepared nurse researchers (DS and PS), one doctorally prepared public health researcher (KD), and a clinical nurse (WG). The team analyzed the data gathered from interview narratives using grounded theory methods (Charmaz 2014), first by reading the transcripts and then by coding word-by-word, followed by categorization. Emerging findings were discussed during team meetings, and all methodological and analytic decisions were documented in an audit trail. No evidence of recall bias was found when comparing the transcripts from four YAs who participated in both studies.

The Young Adult Advisory Group, consisting of four YA daughters whose parents died when they were adolescents, contributed to the study by discussing the findings with DS and PS. Their comments and suggestions were included in the analysis revisions.

“Important for hospice providers to know” emerged as a category, and the research team decided that an in-depth analysis of this topic was warranted. Data pertaining to the category were extracted as individual text units by each member of the team before discussion at data analysis meetings. DS and WG independently reviewed individual text units as extracted by the research team.

The analysis was refined by DS and WG using reflexive thematic analysis methods (Braun and Clarke 2019). They reread the transcripts several times to gain a sense of each YA’s experience. They documented their written reflections after reading each transcript and outlining their thoughts about the individual narratives. Then, they manually coded the data, detailing inductive descriptive codes by marking similar phrases or words from the YAs’ transcripts and generating themes from the codes. The participants’ words were used as labels when possible.

**Figure 1.**
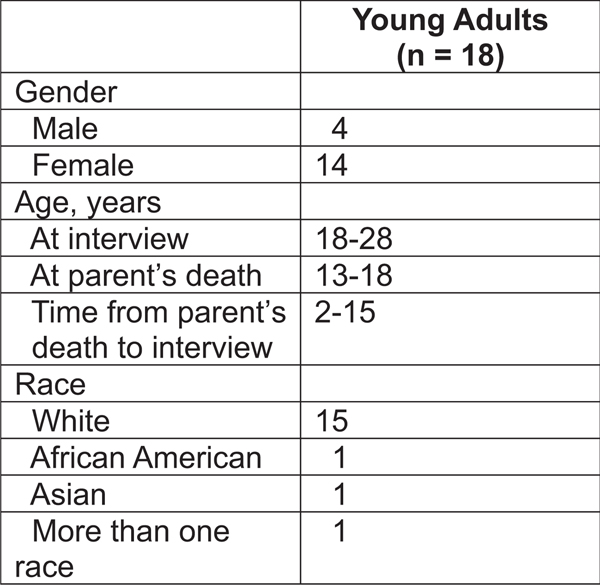
Participation Characteristics.

Several procedures were used to enhance the trustworthiness of our findings. The analysis was conducted by an interdisciplinary research team that included nursing and public health professionals. Peer debriefing occurred after each interview, and discussion of emerging findings took place during team meetings. All methodological and analytic decisions were documented in an audit trail. The sample is described with detailed demographic characteristics (Figure 1), thereby enabling readers to determine whether the findings are relevant to a particular group.

An analysis of the transcripts revealed four themes: Taking the Lead, Listening to Parent and Adolescent Communication Preferences, Providing Resources and Support, and Continuing Support after Parental Death.

## Results

### Taking the lead

The participants clearly identified the need for someone from the hospice team to take the lead in communicating with them. They recommended identifying a team member specifically assigned to the adolescent in the family, potentially a YA whose parent died when he/she was an adolescent; this YA could be a member of the bereavement team.

Having one hospice provider serve as a liaison between the hospice team and the family may be a useful strategy, especially regarding the initial stages of care and distribution of information. We have labeled this strategy “taking the lead.” A 28-year-old daughter stated,

I feel like there was just like, nobody. Somebody needed to come together and consolidate the information and get everybody on the same page to come and get the information. To me, I felt like a lot of information just changed hands and there clearly must have been some disagreement with someone about telling me my mom was going to be terminally ill or something like that.

A hospice provider taking the lead to assess what each family member needs could help minimize uncertainty in the way information is distributed to family members.

When a parent is enrolled in a hospice program, their adolescent children are interested in understanding the purpose of hospice, as some adolescents may not have a working knowledge of what hospice care entails. A 23-year-old daughter explained that hospice providers would give them some information, while the adolescent struggled to grasp the concept of hospice care. She stated,

“They would kind of break it down for us, but I mean at my age I think I mean now we understand the terms and stuff they use but like I mean back then it was like what does any of this mean?” She was 14 years old when her parent was in hospice. A 28-year-old daughter expressed a similar desire for someone to confide in at the beginning, stating “I think if somebody had come in at the beginning of hospice and introduce themselves to my brother and I and made it a point of being there from the beginning, I think I would have been much more inclined to talking to somebody, versus having somebody kind of come in at the end in the last couple of weeks to be there to talk to.” A 26-year-old daughter who was 15 years old when her parent was in hospice shared “the only person I knew on the hospice team was the hospice nurse, who would come in and take care of my dad. And in general, I think we somewhat tried to avoid each other because that’s when my dad was getting baths or diaper changes or things as a kid that I was just providing privacy. And it was my impression that the hospice nurse was *not* there to care for me but was there to care for my dad. I think if someone from the hospice team was – if it was clear to me that part of their role was to talk to me, then I would have been ok with it.”

These quotes present an opportunity for the hospice care team to establish a line of communication with adolescent children from the time care is established to minimize the adolescents’ struggle in determining “what does any of this mean?”

### Listening to parent and adolescent communication preferences

The YAs expressed specific communication preferences among the hospice providers, parents, and adolescents. They suggested a “point person” who would consistently meet with the family. This strategy may be helpful to ensure all family members receive the same information and understand the plan of care. This could also present an opportunity to prepare the family for what is to come throughout the duration of hospice care. It is important for hospice providers to understand the communication preferences of both the parents and adolescents when determining the best way to communicate with the family.

One factor that we discovered during this study that greatly impacts the hospice team’s ways of interacting with adolescent members of a family is communication styles that may be unique to the family. A 28-year-old son shared his insights into his family’s communication patterns “I think it might have been helpful to sit down with somebody as a family myself, my mother, my sister, my brother, maybe even my grandma; sit down as a group and just discuss kind of where everything was at in the process and just kind of left nothing up to imagination or speculation that way we could prepare ourselves for the inevitable a little bit earlier, I guess and maybe I think even start the grieving process at that point even if it’s a couple weeks ahead of time, when you know that they’re obviously in dire shape and they’re not going to be around for, you know, more than a couple more weeks.” This participant’s family preferred to communicate as a unit, rather than having one person relay information to other family members. A 20-year-old daughter said “The way that parents want to share information or not share information with their kids depending on their age and who wants to know what. I think it could be important to try to maybe have the hospice team get a sense of what the parents want to share with their child and how they want to like present the situation so that maybe the child could feel more comfortable.” A 20-year-old daughter explained, “I really did appreciate that they listened to my mom when she said just let my kids be because watching them [hospice providers] interact with my mom we didn’t really mind. I always felt like people were treating me and my brothers like we didn’t understand the situation, that we didn’t know what was going on and the whole pity party of it. We’ve been dealing with it for five years. We go to the doctors’ appointments; my mom explains it [to us].” A 28-year-old daughter explained, “There was never any information being withheld. They were always very, very forthcoming about what was going on. And as my parents got updates from the hospital, we got updates from my parents. There was never a communication gap, which overall I would say makes a huge difference to the adolescent that is going through the experience to hear it straight from your parents’ mouths. To hear exactly what they are hearing from the doctors and to understand how serious it is.” Several YAs talked about structural barriers to interacting with the hospice providers. A 26-year-old daughter commented, “We tried to avoid each other, is what it felt like. I don’t know that that was entirely intentional, other than he [hospice provider] came when I was at school or right around when I came home, and the services he was providing to my dad – I just made myself scarce and tried not to get in the way.” A 20-year-old daughter explained, “I think they [hospice] would come when we are at school, I don’t know if they came on the weekends. I feel like I probably just said hi and like I didn’t really do anything.” These quotes encapsulate the idea that the hospice provider has an important role in assessing individual and family needs regarding communication styles and strategies.

In summary, hospice care providers have a critical role in assisting parents who want to communicate with their children directly but do not know how, for example, guiding parents about responding to difficult questions that they may have themselves (e.g., why is this happening to us). It is important for hospice care providers to consider the communication needs of each family when determining how to share and discuss information about the care and eventual death of a parent.

### Providing support and resources

Adolescent communication styles and support needs also differed among participants. Some adolescents may benefit more from a closely involved hospice provider or therapist during the time their parents are in hospice, while others may benefit more from interventions after the parent’s death. Some adolescents may not require any intervention from the hospice care team at all. A 22-year-old daughter expressed her desire for more interventions after her parent’s death, “I think that talking to someone [on the hospice team] might have been something that I would have utilized more, maybe after his time in hospice care, after he passed. I think scheduling at a time that’s so hectic emotionally would be hard. I wouldn’t want to add any other layers of guilt if I didn’t feel up to it at the time, or stress that what I had scheduled didn’t align with my emotions, so I think as needed would have been really nice, so I was on my own terms when I could [meet with someone].” This quote shows that the participant understands that there are many considerations of both the hospice care team and the adolescent during the time their parents are in hospice, and explains their hesitancy to use resources for themselves, when they feel those resources may be needed elsewhere, specifically for the patient and spouse. A 20-year-old daughter discussed how the hospice team may need to approach each family differently. She said “if you can’t accept help, it might be harder for the hospice team to maybe offer help if they know that they might need to present it in a different way. A lot of people don’t want to feel like they’re being treated any differently just because of the circumstances.”

In most cases, direct interaction and communication between the hospice care team and adolescents is beneficial and leads to adolescents feeling more at ease. This is illustrated by a quote from a 20-year-old son “[The hospice nurse] was talking to me and the way that she made me feel even though I didn’t necessarily explain to her everything that was going on. Just the comfort and the level that she provided. And knowing that that was her job and that she was doing a good job at it just made me feel good. I knew that I could talk to her and almost like I was supposed to.” A 28-year-old daughter who was 13 when her parent was in hospice wanted more information, “I wish they had been a little more honest at the beginning cuz I felt like my understanding of when my mom was terminally ill and the timeline when she was diagnosed terminally ill was not the same. I really think that adults around me should have explained that better cuz I do feel like that once I realized that information was delayed or withheld from me, I felt maybe betrayed. I don’t know how to describe it, but I felt like it was like a betrayal, and I felt kind of like really dumb for not realizing it myself. I had some mental health issues as a teenager and I feel like if I had been put in therapy right away instead of thinking, oh, you know, kids are resilient, she’ll be fine. I feel like that would have helped and I feel like it would have definitely helped me to feel the feelings upfront and not feeling them six months into the diagnosis.” The YAs wanted more information on their parents’ progression. A 22-year-old daughter who was 18 when her parent was in hospice explained, “Something that I thought was really helpful and something that hospice did help me with, was just knowing what to expect in that kind of process, to speak candidly it’s, you don’t really know what to expect with the human body as it goes downhill so it was nice that they kind, they upfront told us these are kind of steps or stages that my parent would follow as they begin to like die and eventually passed. That was super helpful just to kind of know what was going on.”

The YAs shared their thoughts about feeling supported. A 20-year-old daughter who was 18 when her parent was in hospice said “I would say it’s very important just to make sure these adolescents have a support system to talk to, and I feel like that’s very important. I feel like coping has a lot to do with communicating about your feelings and if this adolescent doesn’t have the support system that coping might be a lot harder, and I feel like having a communication and support system is very important when going through a time like that.”

### Continuing support aker parental death

After the death of a parent, the hospice care team’s support for an adolescent should continue. The Medicare Hospice Conditions of Participation (COP) mandates that bereavement services are provided prior to and up to 1 year following the death of the patient based on the family-centered plan of care although these services are not reimbursed. Hospices may need to contract with other community resources to ensure compliance with this regulation (NHPCO Compliance tools and resources Medicare Hospice Conditions of Participation – Bereavement 2022).

It is important to ensure that each family member can cope with the passing of their loved one and has the skills needed to communicate effectively. A 22-year-old daughter expressed her desire for someone from the hospice team to remain as a resource for them after their parents’ death by saying “They could help just mentally understanding the emotional aftermath of losing a loved one.” Further emphasizing the idea that hospice providers should continually assess the needs of a family; a 28-year-old daughter noted “Hospice caregivers should be aware of if you are involved in their caretaking because that also impacts your response to their passing too. If you’re very engaged in their caregiving, especially if it’s a long-term illness like six months to a year plus, the death is going to impact you in a different way than it would if it was a quick death, or you weren’t involved in the caregiving at all.” These statements support the idea that it is important for the hospice provider to “take the lead,” be available to counsel the family, and provide support throughout the duration of hospice care and after the parent’s death.

## Discussion

This study examined the complex inter-relationships among parents in hospice, their adolescent children, and hospice clinicians. The findings suggest strategies that could enhance the care hospice clinicians provide to adolescents from the perspectives of YAs whose parents died 2−15 years prior to the interviews. The quality of the support provided to the adolescents was impacted by their distress when their parents were in hospice and after the parents’ death.

Our results substantiate the findings of prior research indicating that most adolescents do not discuss their feelings about their parents’ hospice care with peers or classmates at school, which could create a space for hospice providers to be a confidant to the adolescent (Sveen et al., 2016). Our results support Fearnley and Boland’s (2019) findings that some hospice clinicians do not perceive that it is their role to deal with psychological concerns and are therefore less likely to encourage communication with hospice patients about their children. The participants in this study identified the need for a consistent point person on the hospice care team to discuss their concerns and provide information and support, which may encourage more trust and consistent sharing of their feelings with the hospice provider.

Listening and paying attention to parent and adolescent communication preferences is imperative to providing personalized support. Our results resonate with findings from other studies. The theme we describe as Listening to Parent and Adolescent Communication Preferences supports Mayo et al.’s (2016) four types of interactions between the adolescents and the hospice care team: no interactions, in-passing interactions, engaged interactions, and formal interactions. Hanna et al. (2021) suggest that healthcare professionals ask their patients about important relationships with their children. When sharing sensitive and often emotionally charged information, it is important for hospice providers to identify the family’s patterns and preferences for communication and to listen closely to both parents and the adolescents. Alvariza et al. (2017) found that teenagers want to be seen and acknowledged during their parents’ illness. They desire information to prepare for the outcomes of their parents’ illness, treatment, and impending death.

A systematic review conducted by Hanna and colleagues (2019) revealed that parents want and need information and guidance from healthcare professionals about a parent’s poor prognosis, especially when death is imminent, so they can prepare their children. Supportive friends, peers, neighbors, relatives, and community groups such as the school were found to be supportive of parents and children when a parent is at the end of their life. A later study conducted by Semple et al. (2021) confirmed these findings. Hanna et al. (2021) reported several barriers to parents sharing prognostic news with their children including a lack of understanding of the parents’ prognosis and feeling ill-equipped. Engagement with social networks, including extended family relatives and peers, and maintaining routines such as attending school were suggested to be supportive by parents and children. Sheehan et al. (2019) found that a parent’s ability to understand the adolescent’s individual needs allowed for more thoughtful interactions.

Semple et al. (2021) recently published a communication framework, Talking, Telling, and Sharing: EOL, which provides a guide for healthcare providers to engage with patients proactively to promote open, family-centered communication when a parent is at the end of their life. Hanna and Semple (2024) found that professionals learned new ways to enhance EOL conversations with parents despite familial resistance to sharing the reality of the situation with their children. The bereaved parents’ lived experience, communication framework, and role-play videos positively shaped clinical practice. The findings from a recent systematic review revealed that healthcare professionals often lack the knowledge, skills, and confidence to best support families when a parent of dependent children is at the EOL. They purport that there is an imminent need for robust educational interventions to be developed, potentially leading to better mental and physical outcomes for the family at the EOL and in bereavement (Sheehan et al. 2023).

Our results support studies that found that adolescents need continued support after their parents’ death (Mayo et al. 2016; Punziano et al. 2017; Semple et al. 2022) and they experience the grieving process in various ways (Farella Guzzo and Gobbi 2021). A healthcare professional reported that a relationship built on empathy, trust, and constant communication was essential for working with the family and the adolescent. They found in their clinical practice that adolescents who were informed about their parents’ disease and involved in their care had more positive responses to their parents’ death and accepted the loss more easily while adolescents who were excluded from these conversations manifested severe reactions of crying and were more traumatized (Punziano et al. 2017).

Franklin et al. (2019) purport a need for healthcare organizations to promote a learning culture to support staff in managing their emotions, facilitating connecting with parents and with children surrounding the death of a parent. Semple et al. (2022) suggested that family-centered bereavement support groups should be offered to the bereaved parent and children to provide support and practical guidance soon after the death of a parent. In addition, peer support can normalize the grief experience while facilitating hope for the future. Mayo et al. (2016) found that bereaved adolescents bonded with peers who had had similar experiences of loss.

Our results provide robust examples of four topics that are important for providers to know when working with adolescents with a parent in hospice: Taking the Lead, Listening to Parent and Adolescent Communication Preferences, Providing Resources and Support, and Continuing Support after Parental Death. Our findings suggest that hospice providers and other clinicians who work with families in which one parent is at the end of their life need to appreciate the family’s unique relationships, situations, and communication preferences when interacting with these adolescents.

While the typology presented here needs further development, the findings have implications for hospice providers who are committed to a family-centered approach to hospice care. The findings indicate, for example, that hospice provider visits need to be scheduled later in the day when teens are more likely to be at home. The hospice provider should recognize that brief interactions during a visit to the parent can affect adolescents and be aware that teens notice the ways in which the hospice provider interacts with their parent. It is important for the hospice provider to be approachable to teens, even when they are focused on providing care to the parent, so that positive in-passing interactions may pave the way for engaged interactions. The hospice provider might also schedule an initial “meet and greet” with the adolescent to circumvent the notion that the providers are there only to care for the ill parent. Asking parents in advance about the teen’s interests and activities can aid in identifying some common ground to improve the chances of making a connection. More training is needed for hospice providers to address the emotional and psychological needs of this population within the context of the family unit.

The IOM’s (2015) definition of hospice care is “person-centered family-oriented care provided by an interdisciplinary team designed to support the person and the family as central members of the team.” Our findings suggest that adolescents, despite being highly valued by the ill parent and with a role in the care of their parents and siblings, are not always recognized as central members of the team with a role.

## Limitations

Several limitations provide context for our findings. Recall bias may have been introduced with participants’ retrospective perceptions of their experiences when their parents were in hospice, although we found no evidence of this in the transcripts of the four YAs who participated in both studies. Because this was a self-selected sample, there may be some bias in favor of those who saw value in talking about their experiences. The extent to which the findings are transferable to other populations is constrained by the limited diversity in our sample.
